# EGCG Alleviates H_2_O_2_‐Induced Inflammatory Injury and Apoptosis in Bovine Mammary Epithelial Cells Through Nrf2 Pathway Activation and p38MAPK Pathway Inhibition

**DOI:** 10.1002/fsn3.4687

**Published:** 2025-02-25

**Authors:** Xuehu Ma, Chunli Hu, Yanhao An, Xue Feng, Peipei Cao, Yun Ma, Yanfen Ma

**Affiliations:** ^1^ College of Animal Science and Technology, Key Laboratory of Ruminant Molecular and Cellular Breeding of Ningxia Hui Autonomous Region Ningxia University Yinchuan China

**Keywords:** antioxidant activity, bovine mammary epithelial cells, epigallocatechin‐3‐gallate, nuclear factor erythroid 2‐related factor 2, p38 mitogen‐activated protein kinase

## Abstract

Epigallocatechin‐3‐gallate (EGCG) is a potential antioxidant that protects cells from oxidative damage. However, EGCG is less studied in oxidative stress‐induced inflammation in bovine mammary epithelial cells (BMECs). Therefore, the present study sought to investigate the protective effects of EGCG on hydrogen peroxide (H_2_O_2_)‐induced oxidative stress, inflammation, and apoptosis, and related mechanisms involved in BMECs using the H_2_O_2_‐induced BMECs as an in vitro cell model of oxidative stress and inflammation response. The BMECs were treated with H_2_O_2_ (600 μM) and EGCG (5 μM), respectively, while the cells without any treatment were regarded as the controls. The protective effects of EGCG were investigated by quantitative real‐time fluorescence PCR, western blot, ELISA, CCK‐8, and so forth. The results showed that the treatment of BMECs with H_2_O_2_ significantly decreased the anti‐oxidation ability of the cells, increased the expression of inflammation‐related factors, and induced apoptosis. Furthermore, the functional recovery test showed that EGCG significantly improved the resistance to oxidative stress, inflammation, and apoptosis in H_2_O_2_‐induced BMECs. The study of the protective mechanisms of EGCG in BMECs showed that EGCG could enter the nucleus by activating nuclear factor erythroid 2‐related factor 2 (Nrf2) and exert the effects of anti‐oxidation and anti‐inflammation upon treatment with BMECs alone. The Nrf2 knockdown assay (siNrf2) showed that siNrf2 upregulated the mRNA expression of inflammatory factors and apoptosis‐related genes in BMECs, increased reactive oxygen species (ROS) accumulation and mitochondrial damage, and downregulated mRNA expression of antioxidant genes. Similarly, EGCG reduced ROS production in BMECs by inhibiting p38 mitogen‐activated protein kinase (p38MAPK) phosphorylation, thereby reducing the mRNA expression of related genes in the NF‐κB/caspase‐3 pathway when p38MAPK was inhibited with the p38MAPK inhibitor SB203580. Overall, the experimental results showed that EGCG could improve the antioxidant function of BMECs by activating the Nrf2 and inhibiting the p38MAPK pathways, reducing inflammation and mitochondrial damage. This study provides a theoretical basis for further study of exogenous EGCG to prevent mastitis in dairy cows.

## Graphical abstract

The present study sought to investigate the protective effects of epigallocatechin‐3‐gallate (EGCG) on hydrogen peroxide‐induced oxidative stress, inflammation, and apoptosis, and related mechanisms. The test showed that EGCG could improve the antioxidant function of bovine mammary epithelial cells by activating the Nrf2 and inhibiting the p38 mitogen‐activated protein kinase pathways, reducing inflammation and mitochondrial damage. This study provides a theoretical basis for further study of exogenous EGCG to prevent mastitis in dairy cows.

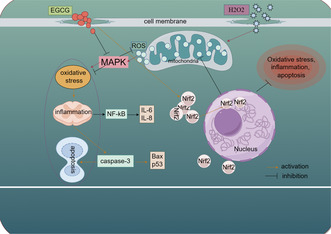

## 1 Introduction

Mastitis in dairy cows is a tissue‐resistant inflammatory response to pathogenic microorganisms‐induced infection of the mammary gland. Currently, mastitis in dairy cows has developed into one of the most prevalent diseases in dairy farming, causing huge economic losses to the dairy farming industry (Wellenberg et al. 2002). Bovine mammary epithelial cells (BMECs) are the first line of defense of cows against environmental attacks (Bruckmaier and Wellnitz 2017). Previous studies have shown that bacteria can secrete many stress proteins to protect themselves from a range of unfavorable environmental stresses (oxidative stress, antibiotics, pH, osmolality, etc.) (Bremer and Kramer 2019; Chiang and Schellhorn 2012; Fernandez and Hancock 2012; Hindupur et al. 2006; Xu, Zhao, et al. 2020). Generally, bacterial infection triggers an innate immune response (Skelton et al. 2019; Zheng et al. 2016). When a host is infected by a pathogen, the host's immune cells phagocytose the pathogen, and the macrophages and heterophilic bacilli may respond to the pathogen oxidatively (El‐Benna et al. 2016; Herb and Schramm 2021), leading to rapid production of reactive oxygen species (ROS) (superoxide anion [$O_{2}^{-}$], hydrogen peroxide [H_2_O_2_], and highly active hydroxyl radical [‐OH] plasma). Subsequently, many cellular sites are destroyed, such as iron–sulfur clusters, cysteine and methionine protein residues, and DNA, eventually leading to cell senescence and death (Hong et al. 2017, 2019; Shivaprasad et al. 2021). Typically, the excessive production of various types of intracellular ROS results from various stressors that may harm the cells and trigger various pathological diseases (Soberanes et al. 2006). Among the ROS produced by various cell types, H_2_O_2_ plays an indispensable role from a physiological point of view as it is produced during normal cellular physiological processes, including oxidative metabolism.

The nuclear factor erythroid 2‐related factor 2 (Nrf2) signaling pathway is an important regulatory pathway for redox reactions. The NF‐κB is a key inflammatory pathway in vivo, which is transferred to the nucleus upon stimulation and promotes the release of inflammatory genes, thereby promoting the production of TNF‐α, IL‐6, and IL‐1β (Carvalho et al. 2013; Lee et al. 2011). It is reported that the activation of the Nrf2 signaling pathway could inhibit the NF‐κB activity and attenuate the inflammatory response. However, the inhibition of Nrf2 could abolish this effect, suggesting that the Nrf2 signaling pathway is associated with the NF‐κB‐mediated inflammatory responses (Buelna‐Chontal and Zazueta 2013; Carayol et al. 2006). In a previous study, the protective effect of Nrf2 against drug‐induced nephrotoxicity was demonstrated by pharmacological activation of Nrf2 and knockdown of Nrf2, respectively (Dong et al. 2023). Similarly, our previous study results demonstrated that the Nrf2 signaling pathway can be activated by tea polyphenols to alleviate cellular oxidative stress and inflammation (Ma et al. 2021).

Mitogen‐activated protein kinase (MAPK) is a type of serine/threonine kinase widely distributed in the spinal cord of animals (Lei et al. 2014). The MAPK pathway can regulate gene expression at three different levels, that is, pre‐transcriptional, posttranscriptional, and translational, which are sequentially activated to regulate a range of cellular processes, including inflammatory responses, cell cycle regulation, cell growth, differentiation, and cellular stress in response to the environment (Burotto et al. 2014). The MAPK pathway also can be activated by different extracellular factors stimulation, such as neurotransmitters, cellular stress, and so forth, and has the function of transmitting signals from the cell membrane to the nucleus. Similarly, the p38 MAPK (p38MAPK) can be activated by different extracellular stimuli, such as neurotransmitters, hormones, and cytokines, to transmit signals from the cell surface to specific sites inside the nucleus. Additionally, they can regulate many cell physiological processes, such as growth, proliferation, differentiation, apoptosis, and so forth, and also participate in the malignant transformation of cells and other pathological processes (Zhang et al. 2017). The p38MAPK is a group of highly evolutionary conserved protein kinases essential for cellular adaptation to environmental changes, immune responses, inflammation, tissue regeneration, and tumor formation. As an indispensable member of the MAPK family, the p38MAPK signaling pathway transduces various extracellular signals and generates many inflammatory mediators, including inflammatory cells and inflammatory factors, in an oxidative stress environment. p38MAPK is a Ser/Thr kinase that catalyzes the reversible phosphorylation of proteins in response to different stimuli, such as cellular stress, infection, or cytokines. However, whether epigallocatechin‐3‐gallate (EGCG) can restrain p38MAPK to inhibit the inflammation of BMECs is still unclear.

Recently, natural compounds have emerged as a potential alternative to many hormones and drugs due to their beneficiary effects on the body's health and could avoid the side effects of many hormones and drugs (Sarkar et al. 2016). In recent years, green tea, including its unique polyphenols and catechins, has been shown to have preventive effects against obesity, cardiovascular diseases, and other diseases (Yang and Hong 2013; Yang and Zhang 2019). Studies have shown that catechins possess antiviral properties and exert protective effects against oxidative stress and inflammation‐induced diseases (Kaihatsu, Yamabe, and Ebara 2018; Steinmann et al. 2013; Xu, Xu, and Zheng 2017). Similarly, in other plant extract studies, resveratrol has been found to play an important role in the fight against oxidative stress, inflammation, neurodegenerative, and other diseases (Koushki et al. 2018). Sudanese citrus fruits are rich in phenolic compounds and vitamin C content and antioxidant active substances (Sir Elkhatim, Elagib, and Hassan 2018); and chlorogenic acid alleviates obesity and regulates the intestinal microbiota of high‐fat‐fed mice, which serves as a protective agent for the organism (Wang, Lam, et al. 2019). The primary components of green tea, including EGCG, gallocatechin, and epigallocatechin, are mainly arranged in descending order (Musial et al. 2020). EGCG, the highest proportion of catechins, is closely associated with various in vitro and in vivo health‐promoting activities, such as antioxidant, anti‐inflammatory, antidiabetic, and cardioprotective (Cavet et al. 2011). However, more in‐depth studies on the application and performance of EGCG are still needed. In this regard, the role of EGCG in oxidative damage BMECs was investigated by knocking down Nrf2 and inhibiting p38MAPK, which provided a novel approach for preventing mastitis in dairy cows.

## 2 Materials and Methods

### 2.1 Cell Culture and Experimental Design

After thawing the BMECs, the cells were inoculated and cultured in six‐well cell culture plates with DMEM/F12 complete medium containing 10% fetal bovine serum at 37°C, 5% CO_2_ concentration, and 100% saturated humidity. The treatment was initiated when the cells achieved 70%–80% growth of their capacity. Then, the BMECs were pretreated with a starvation medium for 18 h as a control group; the H_2_O_2_‐treated group: BMECs were pretreated with a starvation medium for 12 h, followed by 600 μΜ of H_2_O_2_ injury for 6 h (Ma et al. 2021); EGCG‐treated group: pretreated with 5 μM of EGCG for 12 h; EGCG and H_2_O_2_ co‐treatment group: BMECs were first pretreated with 5 μM of EGCG for 12 h, followed by with 600 μM of H_2_O_2_ alone for 6 h.

### 2.2 Total RNA Extraction From Cells

The cells were lysed with 1 mL of RNA‐TRIzol. Then, 200 μL of chloroform was added and allowed to stand for 5 min, followed by mixing thoroughly and centrifugation at 4°C 12,000 rpm for 15 min. The upper transparent liquid was sucked into a centrifuge tube, 0.5 mL of isopropyl alcohol was added and then centrifuged at 12,000 rpm for 10 min. Later, the RNA was rinsed with 1 mL of ice ethanol and centrifuged at 12,000 rpm for 5 min, and the upper liquid was discarded and placed on a clean table. Afterward, the enzyme‐free water was added to dilute the RNA, the RNA concentration was labeled with a multifunctional enzyme‐free instrument, and the RNA integrity was evaluated by gel electrophoresis.

### 2.3 Quantitative Real‐Time Fluorescence PCR

The total RNA was extracted from BMECs using the TRIzol kit (Takara, Japan) and then reverse transcribed into cDNA using the Reverse Transcription Kit (Takara, Japan). A 20 μL qPCR system was used in this experiment, including 0.8 μL each of upstream primer and downstream primer, 2 μL of cDNA, 10 μL of PCR mix with fluorescent dye (Mei5, Beijing, China), and 6.4 μL of water, which was mixed and then transferred to a quantizer (Roche CFX96) for quantitative real‐time fluorescence (qRT‐PCR) detection. The detailed information is summarized in Table 1.

**TABLE 1:** Primer sequences.

Gene	Gene bank	Sequences	Product length (bp)	Tm (°C)
Nrf2	AB162435.1	5′ GACCCAGTCCAACCTTTGTCG 3′ GCTTTTGCCCGTAGCTCATC	185	60
P53	D49825.1	5′ GCCCCTCCTCAGCACCTTAT 3′ GCACAAACACGCACCTCAAA	263	60
Cytc	XM_059885515.1	5′ GTTAGCGGGAACTTCTCGGTC 3′ CCAGTCTTGTGCTTGCCTCC	136	60
AIF	NM_173985.2	5′ CCTGAAGCGAATGATGGAGA 3′ TTGGCTGGGAGACCTGTTG	213	60
IL‐6	NM_173923.2	F: CTGGGTTCAATCAGGCGAT R: CAGCAGGTCAGTGTTTGTGG	206	60
IL‐8	JN559767.1	F: ACACATTCCACACCTTTCCAC R: ACCTTCTGCACCCACTTTTC	149	60
IL‐1β	NM_174093.1	F: CAACCGTACCTGAACCC R: GACACCACCTGCCTGAA	193	60
β‐actin	HM768303.1	F: GCTAACAGTCCGCCTAGAA R: GCAGTCATCACCATCGGCAATGAG	406	60
BAX	NM_173894.1	F: GAGATGAATTGGACAGTAACA R: TTGAAGTTGCCGTCAGAA	118	60
Bcl‐2	NM_001166486.1	F: ATGACCGAGTACCTGAAC R: CATACAGCTCCACAAAGG	605	60
MDA	NM_001046269.2	F: TCGGCTGGGCTAGGATGTT R: AGAGCGTTCTCAGACGATGGA	115	60
GSH‐Px	NM_174076.3	F: TGCGAGGTGAATGGCGAGAA R: GGGACCAGGTGATGAACTTAGGG	118	60
SOD	AY971580.1	F: GACAAATCTGAGCCCTAA R: AAGCAGCAATCTGTAAGC	185	60

### 2.4 Mitochondrial Membrane Potential Assay

An appropriate amount of JC‐1 (200 μm) was taken and diluted at a ratio of 8 mL of ultrapure water per 50 μL of JC‐1 (200 μm). Then, 2 mL of JC‐1 staining buffer was added to prepare the JC‐1 staining solution. First, the culture medium was aspirated from the six‐well plate and rinsed with PBS once. Then, 1 mL of JC‐1 staining working solution was added, mixed thoroughly, and incubated in a cell incubator at 37°C for 20 min. Finally, the supernatant was aspirated and washed with JC‐1 staining buffer (1×) twice, and the fluorescence intensity was observed under a fluorescence microscope.

### 2.5 ROS Testing

DCFH‐DA was diluted with serum‐free culture medium at a ratio of 1:1000 to a final concentration of 10 μM. After removing the cell culture medium from the six‐well plate, approximately 1 mL of diluted DCFH‐DA was added and incubated in a 37°C cell culture incubator for 20 min. After washing thrice with serum‐free cell culture medium, the fluorescence intensity was observed and photographed using a fluorescence microscope.

### 2.6 Cell Viability Assay

The BMECs were inoculated in 96‐well plates. When the cells grew to 70%–80%, 0, 0.5, 1, 2, 3, and 5 μL of EGCG at a concentration of 1 mM were aspirated and added to the 96‐well plates (100 μL of primer) to prepare the EGCG‐activated cells at the concentrations of 0, 5, 10, 20, 30, and 50 μM of actinic BMECs, respectively. After treatment with CCK‐8 reagent (10 μL) at 37°C for 1.5 h, the cells were analyzed at 450 nm, and the cell viability was calculated based on the absorbance.

### 2.7 EdU Cell Proliferation Assay

The assay was performed using the EdU‐555 Cell Proliferation Assay Kit (Beyotime, Shanghai). The cells were inoculated into six‐well plates and incubated with an EdU working solution for 2 h. Then, the cells were removed from the culture medium and fixed with fixative at room temperature for 15 min, and then washed with permeabilizing solution, incubated for 15 min, and the permeabilizing solution was discarded. Finally, the cells were incubated with a click reaction solution for 30 min, removed, and then stained with Hoechest33342 and incubated for 10 min at room temperature (18°C–25°C) for fluorescence detection.

### 2.8 p38MAPK Inhibition and EGCG Solution Preparation

The p38MAPK (SB203580) inhibitor and EGCG purchased from MedchemExpress were diluted to 1 mM with DMSO at −80°C and then diluted to 10 mM. The BMECs were inoculated in a six‐well plate, and the inhibitor was added when the cells reached 70% growth. Finally, the cells were placed in a CO_2_ cell incubator at 37°C for 30 min before aspirating the medium.

According to the instructions, 10 mg of EGCG powder was weighed and dissolved in 21.8164 mL of ultrapure water to configure a 1 mM solution of EGCG, which was stored in a −80 refrigerator. According to the calculation, adding 10 μL of this solution to each six‐well plate is the working concentration of the inhibitor when treating cells.

### 2.9 Data Processing and Analysis

The fluorescence quantification results were analyzed using the 2^−ΔΔCt^ method, and the data were expressed as “mean ± standard error.” One‐way ANOVA was performed using GraphPad Prism 8 software (Song et al. 2023), and multiple comparisons were performed using the LSD method, where *p* < 0.01 was considered as highly significant and *p* < 0.05 was considered as significant.

### 2.10 Interference Nrf2

Before the assay, the cultured BMECs were spread on a six‐well cell culture plate, and the cells were transfected when the cell density reached about 60%. The transfection method was performed following the instruction manual of Lipo3000 (Invitrogen, Shanghai, China). The cell medium was changed to OPTI‐MEM (Gibco, New York, NY, USA) serum‐free medium 30 min before transfection, and the A and B solutions were mixed and left for 20 min. The mixed solution was gradually added into a six‐well cell culture plate, gently mixed, and then incubated in a cell culture incubator at 37°C with 5% CO_2_ for 6 h. Afterward, the serum‐containing growth medium was replaced, the cells were collected after 48 h, and the RNA was extracted and subjected to qPCR to determine the transfection efficiency.

### 2.11 Western Blotting Detection by BMECs

The BMECs were lysed with RIPA lysis buffer, and then the proteins were quantified using a BCA kit. The protein samples were spotted and separated using sodium dodecyl sulfate polyacrylamide gel electrophoresis (SDS‐PAGE), and then the gels were transferred to polyvinylidene difluoride membranes (Bio‐Rad, CA, USA). The membranes were fixed with 5% dry milk powder in potassium trihydrogen hydrochloride buffer and incubated with antibodies against p38, p‐p38, IκBα, p‐IκBα, p65, p‐p65, caspase‐3, B‐celllymphoma‐2 associated X protein (Bax), B‐cell lymphoma 2 (Bcl‐2), and β‐actin (at a 1:1000 dilution) at 4°C overnight. Later, these membranes were washed thrice with TBST and then co‐incubated with sheep anti‐rabbit or mouse IgG antibodies at room temperature for 1 h. Protein expression was then visualized using an ECL chemiluminescent agent, and the protein bands were densitometrically analyzed using an image software.

## 3 Results

### 3.1 Concentration and Screening Time for Optimal EGCG Action

**FIGURE 1:**
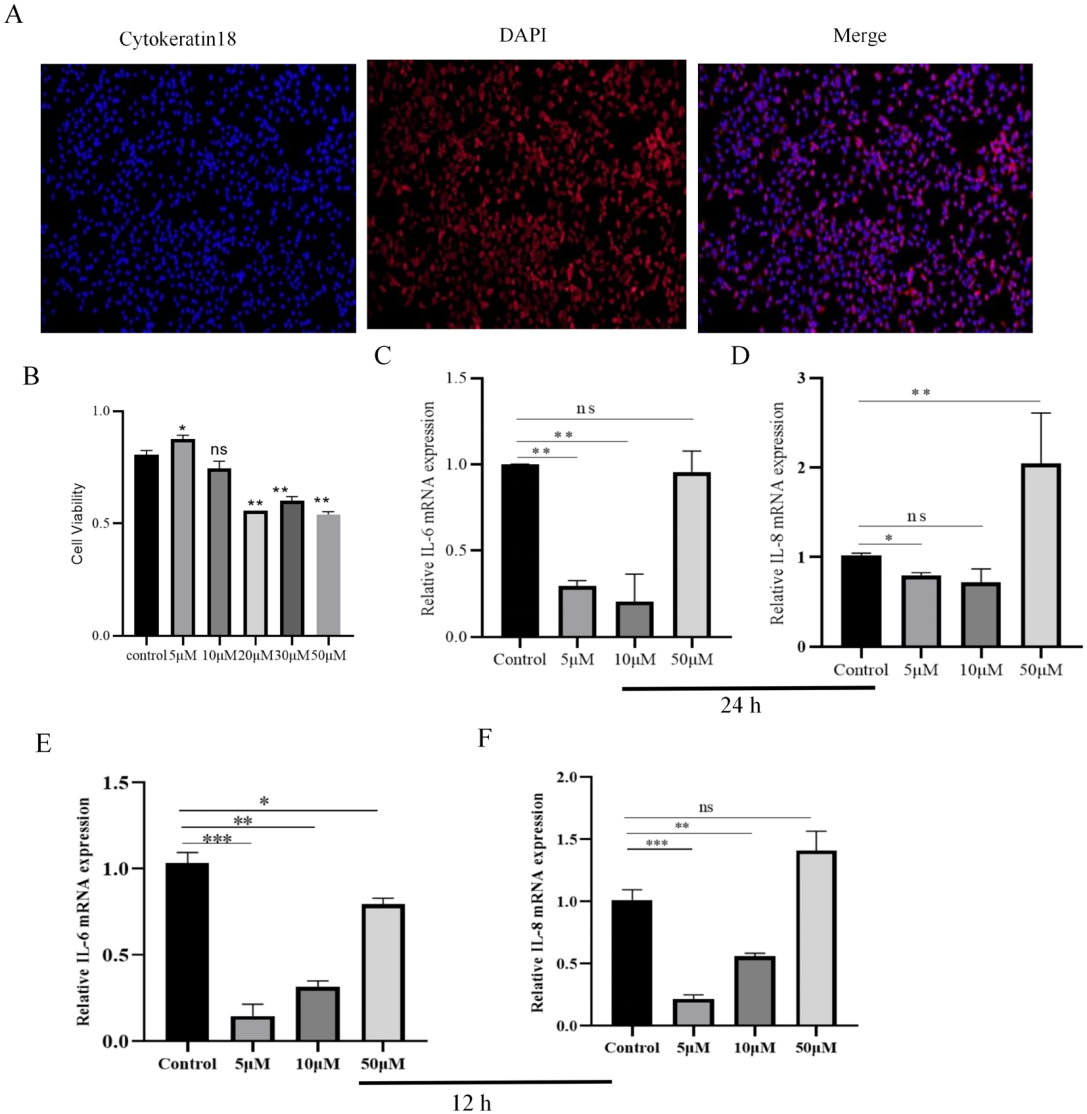
Concentration and screening time for optimal EGCG action. (A) Detection of marker protein (CK‐18) in BMECs lines. (B) The survival rate of BMECs treated with different concentrations of EGCG determined by CCK8. (C) Changes in IL‐6 mRNA expression in BMECs after incubation with different concentrations of EGCG for 24 h. (D) Changes in IL‐8 mRNA expression in BMECs after incubation with different concentrations of EGCG for 24 h. (E) Changes in IL‐6 mRNA expression in BMECs after incubation with different concentrations of EGCG for 12 h. (F) Changes in IL‐8 mRNA expression in BMECs after incubation with different concentrations of EGCG for 12 h. *indicates *p* < 0.05 and ** indicates *p* < 0.01.

DAPI staining and Cy3‐labeled cytokeratin 18 identified the cells used in this assay and showed positive results (Figure 1A), indicating that the cultured cells were BMECs. After that, BMECs were incubated with different concentration gradients of EGCG (0, 5, 10, 20, 30, and 50 μM) for 12 h. The cell viability was detected by CCK‐8 kit, and the optimal concentration of EGCG to protect BMECs was determined. It was found that 5 μM EGCG significantly enhanced the viability of BMECs (Figure 1B, *p <* 0.05), 10 μM EGCG had no significant effect on cell viability (Figure 1B, *p >* 0.05), whereas 20, 30, and 50 μM EGCG significantly inhibited cell viability (Figure 1B, *p <* 0.01). The BMECs were incubated with different concentration gradients of EGCG (0, 5, 10, and 50 μM) for 24 and 12 h, respectively, and the effect of mRNA expression of inflammatory factors IL‐6 and IL‐8 in BMECs was detected by RT‐qPCR, which showed that 5 μM EGCG significantly reduced the mRNA expression of inflammatory factors IL‐6 and IL‐8 at 12 h (Figure 1E,F, *p* < 0.05) and 24 h (Figure 1C,D, *p* < 0.05) significantly reduced the mRNA expression of IL‐6 and IL‐8. Taken together, these results 5 μM 12 h was chosen as the optimal concentration and time of EGCG in this experiment.

### 3.2 EGCG Alleviates H_2_O_2_‐Induced Mitochondrial Dysfunction and Oxidative Stress in BMECs

**FIGURE 2:**
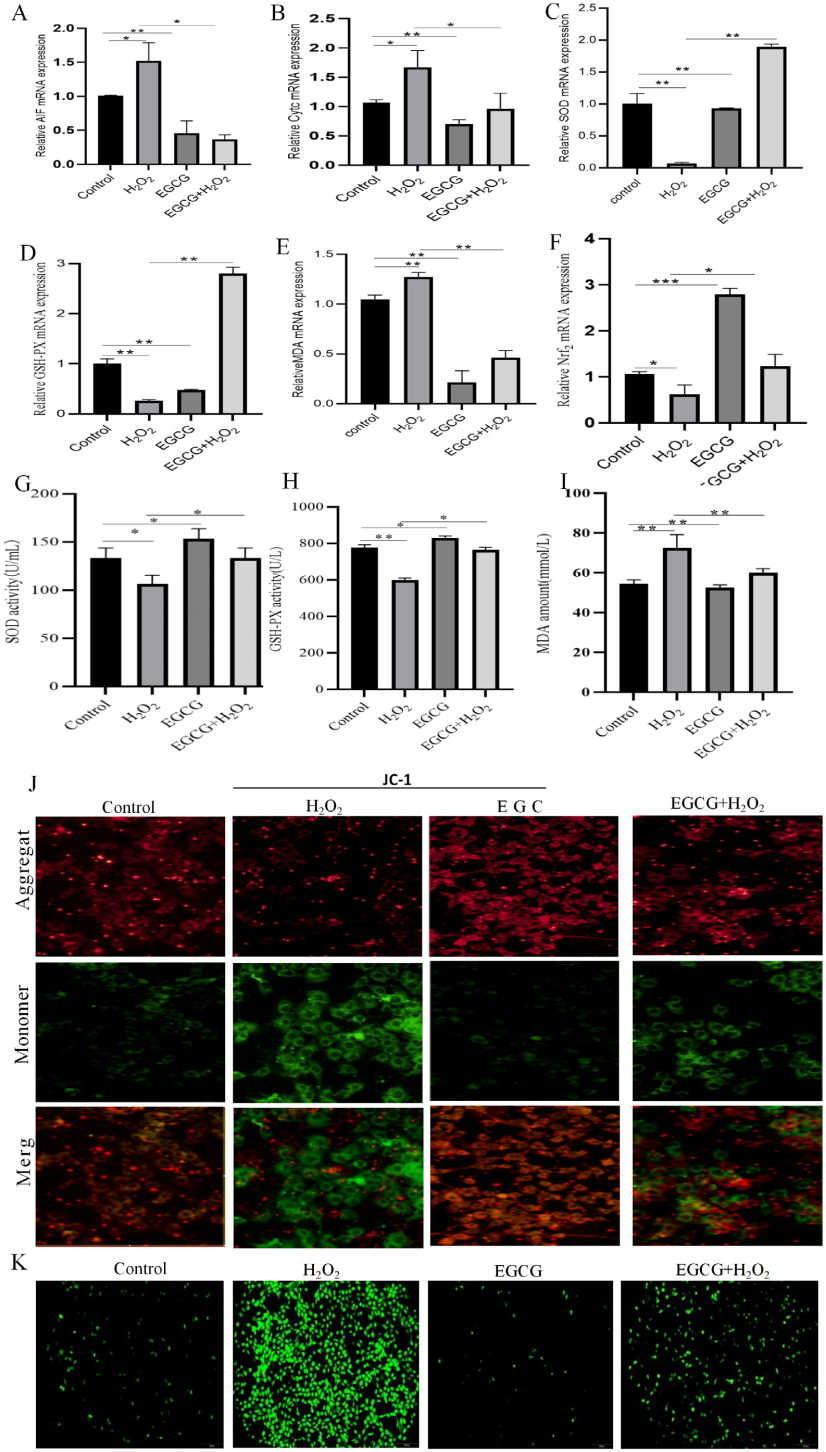
EGCG alleviates H_2_O_2_‐induced mitochondrial function and oxidative stress in BMECs. (A, B) The mRNA expressions of AIF and Cytc in BMECs. (C–F) The mRNA expressions of SOD, GSH‐Px, MDA, and Nrf2. (G, H) The activity of SOD and GSH‐Px in BMECs. (I) The content of MDA in BMECs. (J) Mitochondrial membrane potential changes (200 μm) in BMECs. (K) Microscopic changes in ROS fluorescence in BMECs (200 μm).

Mitochondria‐related indexes were first examined, and the results showed that the mRNA expression levels of apoptosis‐inducing factor (AIF) (Figure 2A, *p* < 0.01) and cytochrome C (Cytc) (Figure 2B, *p* < 0.01) in mitochondria were significantly higher than those in the control group after incubation of BMECs with H_2_O_2_, whereas the mRNA expression levels of them were significantly lower than those of the co‐treatment of EGCG and H_2_O_2_ group (*p* < 0.01). Similarly, mitochondrial membrane potential showed a decrease in the level of mitochondrial membrane potential after incubation of BMECs with H_2_O_2_ (Figure 2J), whereas their fluorescence level was significantly higher after co‐treatment with EGCG and H_2_O_2_ than in the H_2_O_2_ group. Afterward, oxidative stress‐related indexes were examined, and the results showed that when BMECs were incubated with H_2_O_2_, the mRNA and protein expression levels of the intracellular antioxidant factors SOD (Figure 2C, *p* < 0.01; Figure 2G, *p* < 0.01), GSH‐Px (Figure 2D, *p* < 0.01; Figure 2H, *p* < 0.01), and MDA (Figure 2E, *p* < 0.01; Figure 2I, *p* < 0.01) were significantly reduced. Meanwhile, the ROS fluorescence results showed that ROS fluorescence was enhanced after incubation of BMECs with H_2_O_2_ (Figure 2K), while its fluorescence level was significantly lower after co‐treatment with EGCG and H_2_O_2_ than that of the H_2_O_2_ group.

### 3.3 EGCG Attenuates H_2_O_2_‐Induced Inflammation and Apoptosis in BMECs

**FIGURE 3:**
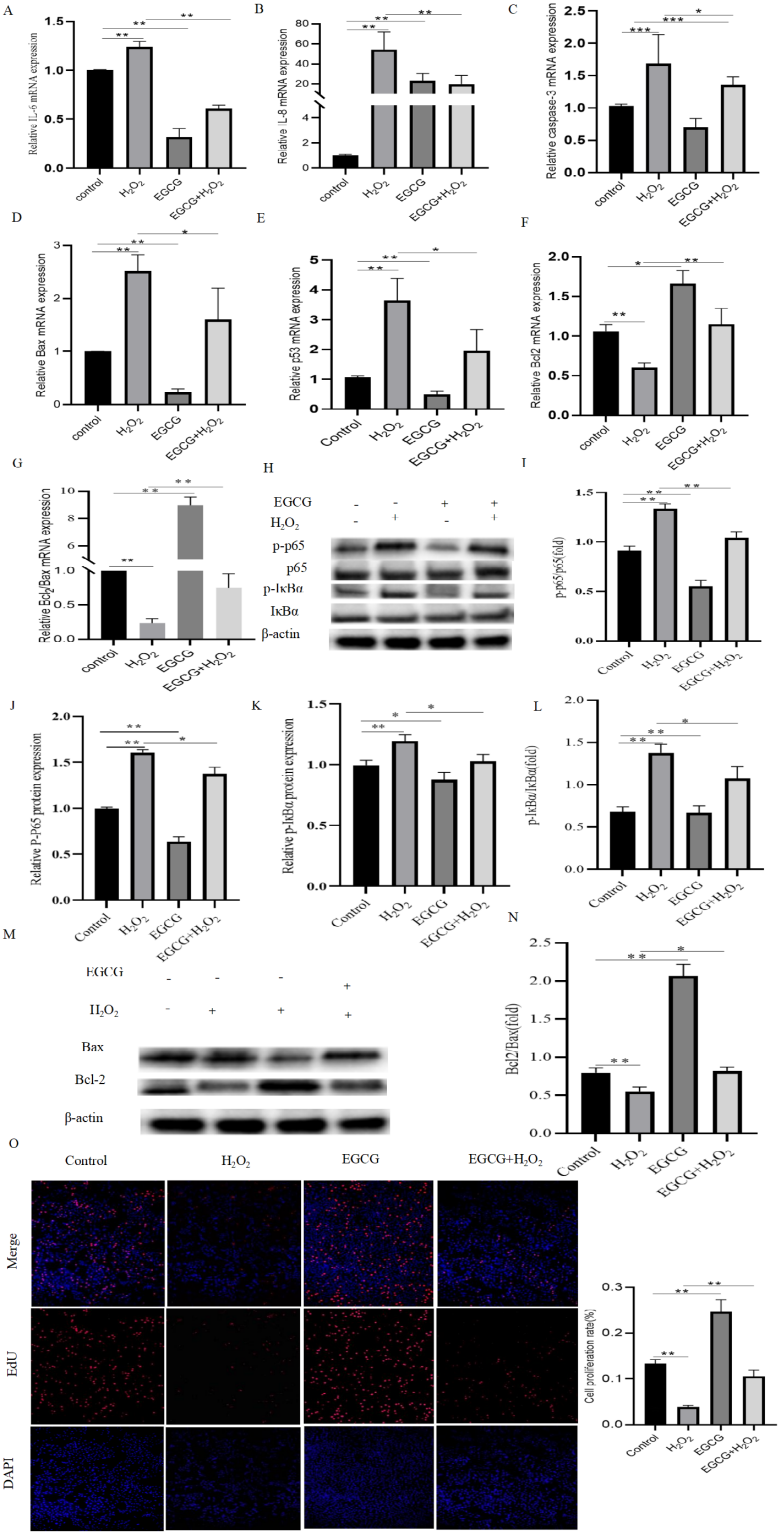
EGCG attenuates H_2_O_2_‐induced inflammation and apoptosis in BMECs cells. (A, B) mRNA expression of cellular inflammatory factors IL‐6 and IL‐8. (C–E) mRNA expression of apoptosis factor caspase‐3, Bax, and antiapoptotic factor Bcl‐2. (F, G) The ratio of dimerized Bcl‐2/Bax and mRNA expression of apoptotic factors p53. (H–J) Protein expression of NF‐κB‐related proteins p‐p65, p65 and p‐IκBα, IκBα in BMECs and their quantitative plots. (K‐N) Apoptotic proteins Bcl‐2 and Bax protein expression and their ratios. (O) EdU cell proliferation bar graph and EdU cell proliferation fluorogram (200 μm).

The mRNA expression of inflammatory factors IL‐6 and IL‐8 was first detected (Figure 3). The results showed that H_2_O_2_ treatment significantly upregulated the mRNA expression of IL‐6 and IL‐8, whereas EGCG or EGCG and H_2_O_2_ co‐treatment significantly decreased the mRNA expression of IL‐6 (Figure 3A, *p* < 0.01) and IL‐8 (Figure 3B, *p* < 0.01) in BMECs. In addition, mRNA expression of apoptosis‐related factors caspase‐3, Bax, and p53, and mRNA expression of the antiapoptotic factor Bcl‐2, as well as apoptotic dimerization Bcl‐2/Bax values were examined. The results showed that compared with the control group, H_2_O_2_ treatment significantly upregulated the mRNA expression of caspase‐3 (Figure 3C, *p* < 0.01), Bax (Figure 3D, *p* < 0.01), and p53 (Figure 3E, *p* < 0.01) and significantly downregulated the mRNA expression of Bcl‐2 (Figure 3F, *p* < 0. 01) in the BMECs, whereas EGCG could significantly downregulate the mRNA expression of mRNA expression of caspase‐3 (Figure 3C, *p* < 0.01), Bax (Figure 3D, *p* < 0.01) and p53 (Figure 3E, *p* < 0.01). Similarly, after co‐treatment with EGCG and H_2_O_2_, the value of Bcl‐2/Bax was significantly higher than that in the H_2_O_2_ group. The protein expression of NF‐κB‐related proteins p65, p‐p65, p‐IκBα, and IκBα was detected by Western blot. The protein expression of p‐p65 (Figure 3J, *p* < 0.01) and p‐IκBα (Figure 3K, *p* < 0.01) was significantly upregulated in the H_2_O_2_‐treated group compared with the control group. In contrast, protein expression of p‐p65 (Figure 3J, *p* < 0.05) and p‐IκBα (Figure 3K, *p* < 0.05) decreased significantly after EGCG and H_2_O_2_ co‐treatment. In addition, we examined the protein expression of Bax and Bcl‐2 and found that Bax (Bax, Figure 3M,N) was significantly decreased and Bcl‐2 (Figure 3M,N) was elevated after co‐treatment of EGCG with H_2_O_2_ compared to the H_2_O_2_‐treated group. The effect of EGCG on cell proliferation was detected using the EdU kit, and its quantitative results showed that EGCG significantly increased the proliferation rate of BMECs (Figure 3O).

### 3.4 EGCG Improves Mitochondrial Function and Antioxidant Capacity of BMECs by Activating Nrf2

**FIGURE 4:**
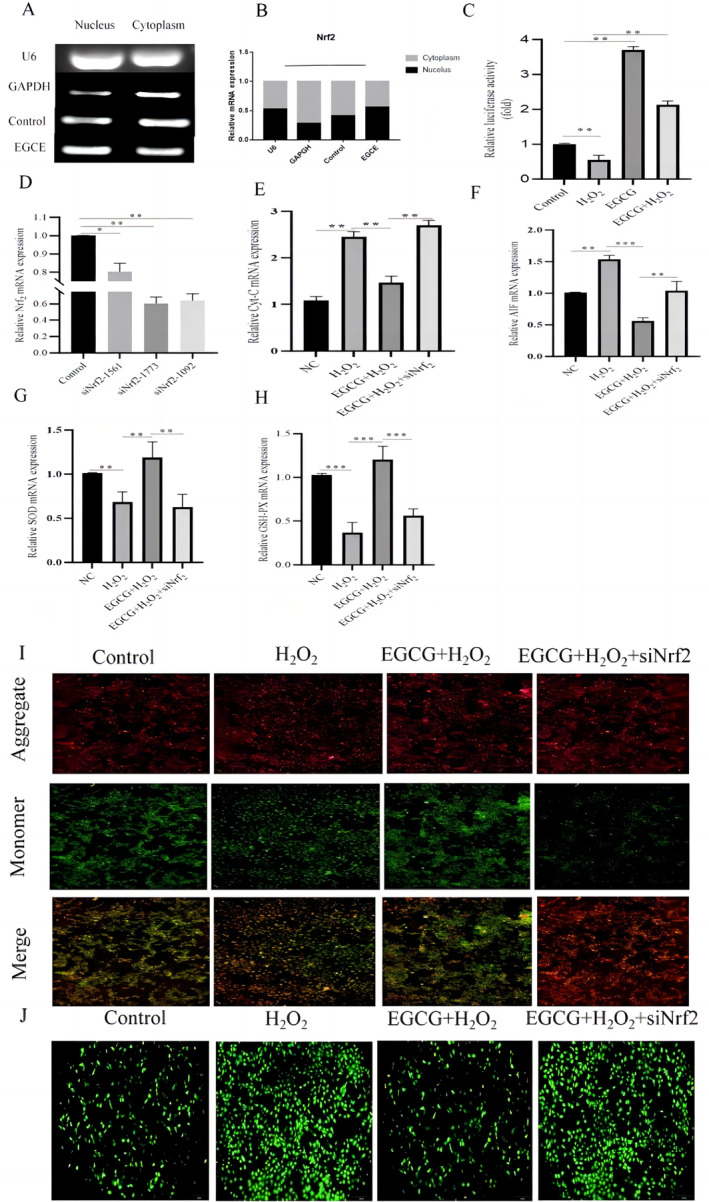
EGCG alleviates oxidative stress and mitochondrial function in BMECs by activating Nrf2. (A, B) Graph of Nrf2 in the nucleus and cytoplasm in the control and EGCG groups in BMECs. (C) Activity of ARE. (D) Efficacy of screening Nrf2 short interfering RNA (siRNA) fragments. (E, F) BMECs mitochondrial mRNA expression of associated factors Cytc, AIF. (G, H) mRNA expression of antioxidant factors GSH‐Px, SOD in BMECs after Nrf2 knockdown. (I) Changes in mitochondrial membrane potential after Nrf2 knockdown. (J) Changes in ROS fluorescence in BMECs after Nrf2 knockdown.

The expression of Nrf2 in the nucleus and cytoplasm was first determined by subcellular localization method. The results showed that the gene expression of Nrf2 in the EGCG‐treated group was significantly higher than that in the control group (Figure 4A,B). In addition, the results of the activity assay of ARE in BMECs showed that H_2_O_2_ treatment significantly decreased the activity of ARE, whereas EGCG treatment or the combination of EGCG and H_2_O_2_ treatment significantly increased the activity of ARE (Figure 4C, *p* < 0.01). The highest interference efficiency of siNrf2‐1773 was subsequently found by screening the interference fragment of Nrf2 (siNrf2) (Figure 4D), which was used in the subsequent interference assay. The results of the interference assay showed that the mRNA expression of Cytc (Figure 4E, *p* < 0.01) and AIF (Figure 4F, *p* < 0.01) was significantly increased in the EGCG and H_2_O_2_ co‐treated group after knockdown of Nrf2. In addition, the mRNA expression of SOD and GSH‐Px after Nrf2 was knocked down was also examined, and the results showed that the gene expression of SOD (Figure 4G, *p* < 0.01) and GSH‐Px (Figure 4H, *p* < 0.01) was significantly downregulated in the EGCG and H_2_O_2_ co‐treated group, but compared with that, the gene expression of SOD (Figure 4G, *p* < 0.01) and GSH‐Px after knocking down Nrf2 (Figure 4H, *p* < 0.01) expression was significantly reduced. The MMP results showed that the membrane potential was restored in the EGCG and H_2_O_2_ co‐treated group, whereas it was significantly decreased in the EGCG and H_2_O_2_ co‐treated group after Nrf2 was knocked down. The fluorescence intensity of the decreased membrane potential was consistent with that of the H_2_O_2_ group (Figure 4I). The ROS fluorescence results showed that the fluorescence intensity of ROS in the cells was significantly enhanced after Nrf2 knockdown (Figure 4J).

### 3.5 EGCG Alleviates BMEC Inflammation and Apoptosis by Activating Nrf2

**FIGURE 5:**
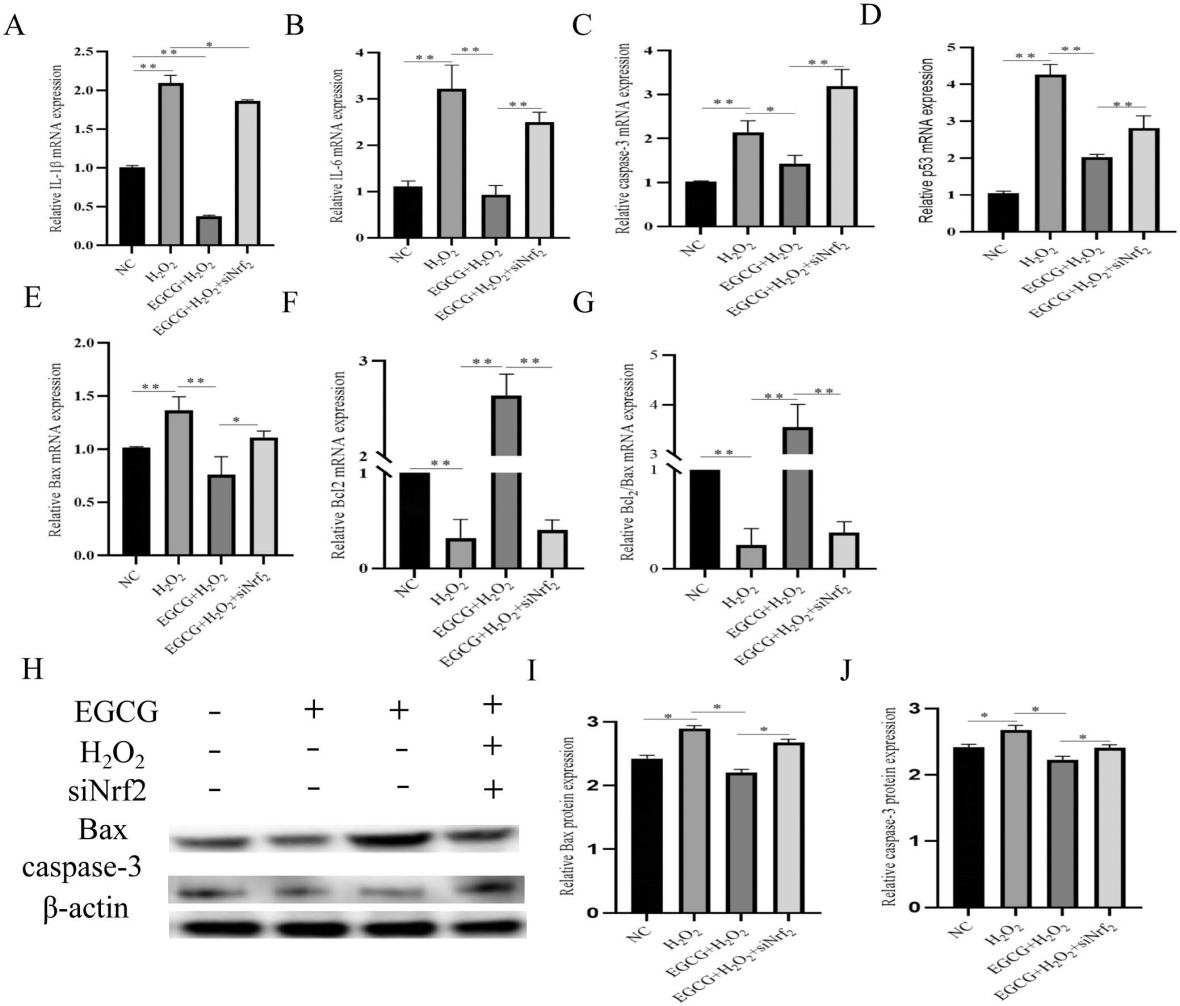
EGCG alleviates BMECs inflammation and apoptosis through Nrf2 activation. (A, B) mRNA expression of inflammatory genes IL‐1β and IL‐6 in BMECs after interference with Nrf2. (C–E) mRNA expression of apoptotic factors caspase‐3, p53, and Bax, in BMECs after interference with Nrf2. (F) mRNA expression of antiapoptotic factor Bcl‐2 in BMECs after interference with Nrf2. (G) Heterodimer Bcl‐2/Bax ratio. (H–J) Protein expression of caspase‐3 and Bax in BMECs after interference with Nrf2 and its quantitative plot.

The gene expression of IL‐1β and IL‐6 in BMECs was examined by qPCR. mRNA expression of IL‐6 (Figure 5A, *p* < 0.01) and IL‐8 (Figure 5B, *p <* 0.01) in the H_2_O_2_‐treated group was significantly higher than that of the control group, whereas the mRNA expression of IL‐6 and IL‐8 in the co‐treated group with EGCG and H_2_O_2_ was significantly reduced, but siNrf2 treatment increased significantly after siNrf2 treatment. Compared with the H_2_O_2_‐treated group, Caspase‐3 (Figure 5C, *p* < 0.01); p53 (Figure 5D, *p <* 0.01); and Bax (Figure 5E, *p* < 0.01) were significantly decreased in the EGCG and H_2_O_2_ co‐treated group, but which increase in concentration at the combination of EGCG and H_2_O_2_ after siNrf2 (Figure 5E), and the mRNA expression of Bcl‐2 was significantly decreased (Figure 5F, *p* < 0.01). In addition, the protein levels of Bax and caspase‐3 were detected by Western blot, and it was found that the protein expression of Bax (Figure 5H) and caspase‐3 (Figure 5H) in the EGCG and H_2_O_2_ co‐treated group was significantly lower than that in the H_2_O_2_‐treated group. However, after Nrf2 interference, the protein expression of Bax and caspase‐3 increased, and the trend of change can be seen directly in the quantitative graphs (Figure 5I, *p <* 0.05; Figure 5, Gcaspase‐3, *p* < 0.05) (Table 2).

**TABLE 2:** siNrf2 RNA fragment sequence.

siNrf2‐1561	5′‐GCAAUUCAACGAGGCUCAATT‐3′ 5′‐UUGAGCCUCGUUGAAUUGCTT‐3′
siNrf2‐1773	5′‐CCUUGUAUCUUGAAGUCUUTT‐3′ 5′‐AAGACUUCAAGAUACAAGGTT‐3′
siNrf2‐1092	5′‐GCACAACAGCAGAAUUCAATT‐3′ 5′‐UUGAAUUCUGCUGUUGUGCTT‐3′

### 3.6 EGCG Mitigates BMEC Injury by Inhibiting p38MAPK

**FIGURE 6:**
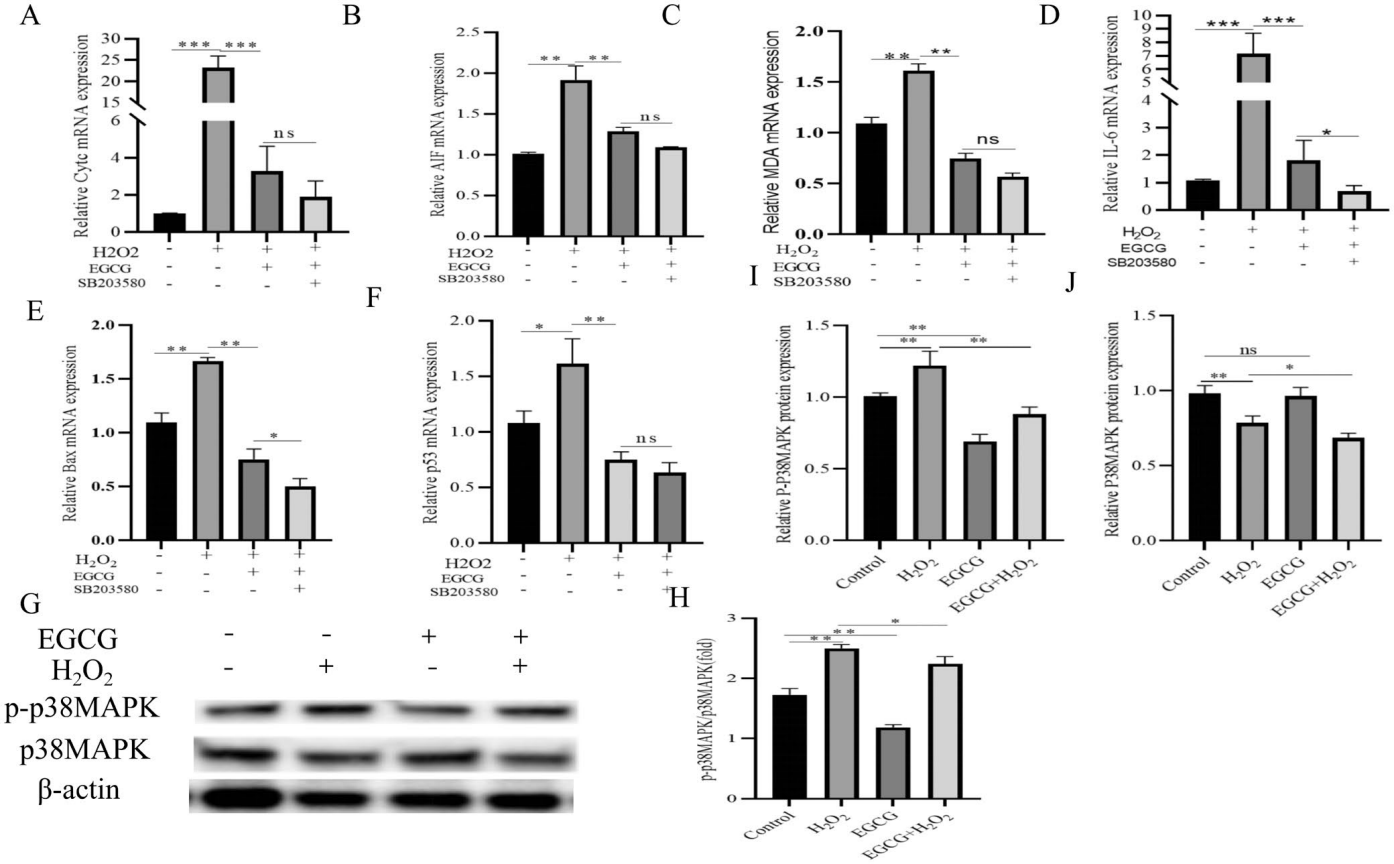
EGCG alleviates BMECs injury by inhibiting p38MAPK. (A, B) mRNA expression of mitochondrial and apoptosis‐related factors Cytc and AIF in BMECs after p38MAPK inhibition. (C) mRNA expression of the antioxidant factor malondialdehyde (MDA) after p38MAPK inhibition. (D) mRNA expression of the inflammatory factor IL‐6 after p38MAPK inhibition. (E, F) mRNA expression of the apoptotic factors Bax and p53 in BMECS after p38MAPK inhibition. (G, H) Changes in p38MAPK protein expression and their quantitative plots.

The inhibitory effect of EGCG on p38MAPK was examined before the use of the inhibitor, and the results showed that H_2_O_2_ significantly increased the protein expression of p‐p38MAPK compared with the control (Figure 6I, *p <* 0.01), whereas EGCG significantly inhibited the protein expression of p38MAPK (Figure 6J, *p <* 0.01). Similarly, co‐treatment of EGCG with H_2_O_2_ significantly attenuated H_2_O_2_‐induced injury in BMECs (Figure 6G–J). The mRNA expression of mitochondria‐associated factors Cytc (Figure 6A, *p >* 0.05) and AIF (Figure 6B, *p* > 0.05) was significantly decreased in the inhibitor (SB203580)‐treated group compared to the co‐treated group with EGCG and H_2_O_2_. mRNA expression of MDA was not significantly upregulated (Figure 6C, *p >* 0.05). mRNA expression of IL‐6 (Figure 6D, *p* > 0.05) and mRNA expression of p53 (Figure 6E, *p* > 0.05) did not change significantly, indicating that EGCG inhibited p38MAPK to exert anti‐inflammatory and antioxidant effects, but the expression of Bax was significantly decreased (Figure 6F, *p* < 0.05).

## 4 Discussion

H_2_O_2_ is a trigger of oxidative stress and inflammation in many cell types (Pinheiro et al. 2022; Zhang et al. 2018; Zhou et al. 2021). It is reported that an increased endogenous or exogenous H_2_O_2_ impairs the immune and free radical elimination capacity of mammary tissue, thereby triggering mastitis (Abalenikhina et al. 2022; Basu et al. 2014). EGCG reduced ROS production and inhibited inflammatory pathways, and apoptotic pathways in this study. Moreover, EGCG exerted protective effects on inflammation and oxidative responses in H_2_O_2_‐induced BMECs by inhibiting the p38MAPK and activating the Nrf2 signal transduction pathways. Additionally, EGCG ameliorated apoptosis induced by H_2_O_2_ oxidative damage and enhanced cell proliferation.

H_2_O_2_ is an extremely unstable reactive ROS, which can easily decompose to generate hydroxyl radicals and cause peroxidative damage to cells (Goldshmit et al. 2001; Kitamura et al. 1999). When the original REDOX dynamic balance in the cell is disrupted, a large number of oxidation intermediates, such as ROS, superoxide anions, or H_2_O_2_ are produced, leading to oxidative stress damage (Sies et al. 2022). Meanwhile, the activities of endogenous antioxidant enzymes, such as catalase, SOD, and glutathione peroxidase, which could remove the oxidation products, are also significantly inhibited (Charradi et al. 2018). In this study, the H_2_O_2_‐induced BMECs showed a dramatic increase in intracellular ROS, leading to a significant increase in the mRNA expression of inflammatory factors (IL‐6, IL‐8), oxidant indicators (MDA), and apoptotic factors (Bax, caspase‐3). Additionally, the mRNA expressions of SOD and GSH‐PX were reduced significantly, which was consistent with our previous study results (Ma et al. 2019). In this study, the antioxidant capacity of cells was significantly improved with the addition of EGCG to BMECs. This result was consistent with the effects of procyanidin B2 and astragaloside IV on heat‐induced and ammonia injury‐induced oxidative stress and apoptosis in vascular cells (Wang, Zhao, et al. 2019; Wang et al. 2022).

Nrf2 is a transcription factor involved in cellular stress response, which enhances the cellular defense response to oxidative stress by regulating the transcription of antioxidant factors. Additionally, Nrf2 plays a vital role in many cellular processes, such as apoptosis, mitochondrial metabolism, and inflammatory responses (Hsieh et al. 2022). When the cell is under oxidative stress, oxidation of the active site of the E3 ligase aptamer Kelch‐like ECH‐associated protein 1 (Keap1) results in the separation of Keap1 from Nrf2, leading to the invasion of Nrf2 into the nucleus (Motohashi and Yamamoto 2004). In this study, the intracellular ROS level increased significantly after Nrf2 was knocked out, resulting in a decrease in the cellular antioxidant capacity and significant downregulation of the activity of corresponding antioxidant enzymes SOD and GSH‐Px. In hepatocyte studies, EGCG was found to enhance the antioxidant capacity of hepatocytes and alleviate liver injury in mice by activating the Nrf2 signaling pathway (Xu et al. 2022). In this study, Nrf2 exerted antioxidant effects in the nucleus through subcellular localization after BMECs were treated with EGCG, which was consistent with the anti‐oxidation, anti‐inflammatory, and antiapoptotic effects of other natural antioxidants upon activation of Nrf2 into the nucleus of cells (Meng et al. 2022; Sun et al. 2020). Activation of the Nrf2 signaling pathway significantly reduced the inflammatory response and inhibited the NF‐κB activity. However, the anti‐inflammatory effect disappeared when Nrf2 and HO‐1 were knocked down, suggesting that the Nrf2 signaling pathway plays an important role in the NF‐κB‐mediated inflammatory response (Buelna‐Chontal and Zazueta 2013; Minelli et al. 2012). Additionally, the mRNA expressions of inflammatory factors and apoptosis factors in BMECs were significantly increased after Nrf2 knockdown, indicating that the knockdown or inhibition of Nrf2 might trigger the inflammatory response of BMECs and the occurrence of apoptosis. This was consistent with the action mechanism of tea polyphenols in our previous study, reporting that tea polyphenols could reduce H_2_O_2_‐induced oxidative damage in BMECs by activating Nrf2 (Ma et al. 2019, 2021).

MMP is a key parameter to evaluate mitochondrial function (Chen 1988), and many diseases are associated with mitochondrial dysfunction (Pieczenik and Neustadt 2007). The experimental results proved that EGCG could protect BMECs from mitochondrial damage caused by H_2_O_2_ by activating Nrf2. Many toxic compounds can reduce MMP by disrupting various macromolecules in mitochondria, thus affecting mitochondrial functions, and the reduction in MMP might be related to apoptosis (Lemasters et al. 2002). Studies have shown that the change in intracellular and extracellular Ca^2+^ concentration balance and intracellular Ca^2+^ overload leads to the change in MMP and the opening of mitochondrial inner membrane permeability pores, resulting in the release of Cytc from the mitochondrial membrane gap (Oltvai, Milliman, and Korsmeyer 1993). Additionally, an increase in ROS after H_2_O_2_ damage cells directly damages the DNA, leading to the activation of oncogene p53 and causing apoptosis (Freire et al. 2021). The cystathionine pathway plays a vital role in controlling the mitochondrial apoptosis pathway, and caspase‐3 is also critical in the apoptosis pathway (Boatright and Salvesen 2003; Pu et al. 2017). Under normal conditions, caspase‐3 exists as an inactive zymogen in the cytoplasm, and when apoptotic signals are sent out, caspase‐3 is cleaved and activated by various protein hydrolases, initiating a cascade reaction that leads to apoptosis (Lockshin 2005; Oh and Lim 2006). Herein, the expressions of apoptosis‐related genes Cytc, p53, caspase‐3, and Bax in mitochondria were significantly reduced after EGCG treatment, while the mRNA expression of antiapoptotic factor Bcl‐2 was promoted. Finally, the apoptosis of BMECs induced by H_2_O_2_ was alleviated after EGCG treatment.

The key protein p38MAPK In the MAPK signaling pathway plays a crucial role in cell migration, apoptosis, proliferation, and differentiation (Teegala et al. 2022). It has been shown that the bioactive substance forsythiaside attenuates LPS damage in mouse mammary glands by inhibiting p38MAPK (Tong et al. 2021). Similarly, the present study results demonstrated that EGCG could inhibit the protein expression of phosphorylated p38MAPK to reduce inflammation and apoptosis. SB203580 has been applied as an inhibitor of p38MAPK in various cell types (Li et al. 2020; Paw et al. 2021; Wang et al. 2021). In a previous study, the activation of the p38MAPK signaling pathway upregulated the phosphorylated p38MAPK expression, thereby inhibiting the proliferation of mouse hippocampal stem cells, while treatment with SB203580 enhanced the proliferation viability of mouse hippocampal stem cells (Tian et al. 2017). In this study, H_2_O_2_ treatment activated p38MAPK, leading to an increase in the mRNA expression of cellular inflammatory (IL‐6) and apoptotic factors (Bax, p53), whereas the co‐treatment of EGCG and H_2_O_2_ significantly reduced the mRNA expression of intracellular inflammatory and apoptotic factors. However, after the activation of p38MAPK inhibitor (SB203580), there were no significant changes in the intracellular inflammatory factors and apoptotic factors compared with the EGCG and H_2_O_2_ co‐treatment groups, indicating that EGCG exerts the anti‐inflammatory and antiapoptotic effects by inhibiting p38MAPK. This was consistent with previous study results, reporting that the Rho family GTPase 2 (RND2) can inhibit autophagy and reduce cell apoptosis by inhibiting the p38MAPK signaling pathway (Xu, Sun, et al. 2020). Besides, aspirin eugenol ester, an antioxidant and anti‐inflammatory agent, has a significant protective effect on paraquat‐induced lung injury in rats by inhibiting ROS/p38MAPK (Zhang et al. 2021). The preliminary results of this study demonstrated that EGCG may regulate the health of BMECs by restoring the mitochondrial function after p38 MAPK inhibition. In conclusion, EGCG can inhibit p38MAPK and alleviate oxidative damage in BMECs by improving mitochondrial function.

## 5 Conclusion

In summary, an increased intracellular H_2_O_2_ concentration leads to mitochondrial dysfunction, oxidative damage, inflammation, and apoptosis in BMECs. However, EGCG can attenuate H_2_O_2_‐induced mitochondrial dysfunction, oxidative damage, inflammation, and apoptosis in BMECs. The anti‐oxidative and anti‐inflammation mechanism of EGCG suggests that EGCG can activate the Nrf2 pathway and inhibit the p38MAPK pathway to enhance the anti‐oxidative enzyme activity and mitochondrial function and decrease the expression of inflammatory and apoptotic factors. Overall, EGCG has the potential to alleviate H_2_O_2_‐induced mastitis in dairy cows. This study provides a novel strategy for the subsequent prevention of mastitis using EGCG.

## Author Contributions

**Xuehu Ma:** conceptualization (equal), formal analysis (equal), writing – original draft (equal). **Chunli Hu:** data curation (equal), software (equal). **Yanhao An:** data curation (equal), software (equal). **Xue Feng:** methodology (equal), validation (equal). **Peipei Cao:** methodology (equal), validation (equal). **Yun Ma:** conceptualization (equal), project administration (equal), supervision (equal). **Yanfen Ma:** funding acquisition (lead), project administration (lead), writing – review and editing (lead).

## Conflicts of Interest

The authors declare no conflicts of interest.

## Data Availability

The data presented in this study are available in the article.
