# Correction to “EGCG Alleviates H_2_O_2_‐Induced Inflammatory Injury and Apoptosis in Bovine Mammary Epithelial Cells Through Nrf2 Pathway Activation and p38MAPK Pathway Inhibition”

**DOI:** 10.1002/fsn3.70202

**Published:** 2025-05-07

Ma, X., Hu, C., An, Y., Feng, X., Cao, P., Ma, Y. and Ma, Y. (2025), EGCG Alleviates H_2_O_2_‐Induced Inflammatory Injury and Apoptosis in Bovine Mammary Epithelial Cells Through Nrf2 Pathway Activation and p38MAPK Pathway Inhibition. Food Sci Nutr, 13: e4687. https://doi.org/10.1002/fsn3.4687

In Funding section, the follow text was incorrect: “This work was supported by grants from the Ningxia Ruminant Nutrition Science and Technology Innovation Team (2024CXTD008, Yinchuan, China); National Natural Science Foundation of China (No. 32060765, Beijing, China); Ningxia Natural Science Foundation of China (No. 2022AAC02006, Yinchuan, China); Ningxia University Graduate Student Innovation Program (CXXM202348); and Ningxia Hui Autonomous Region Top‐notch Youth Talents Project (No. 2022).”

The correct statement is: “National Natural Science Foundation of China (No. 32060765, Beijing, China); Ningxia Ruminant Nutrition Science and Technology Innovation Team (No. 2024CXTD008, Yinchuan, China); Ningxia Hui Autonomous Region Top‐Notch Youth Talents Project (No. 2022).”

We apologize for this error.

